# Accelerated and Improved Vascular Maturity after Transplantation of Testicular Tissue in Hydrogels Supplemented with VEGF- and PDGF-Loaded Nanoparticles

**DOI:** 10.3390/ijms22115779

**Published:** 2021-05-28

**Authors:** Federico Del Vento, Jonathan Poels, Maxime Vermeulen, Bernard Ucakar, Maria Grazia Giudice, Marc Kanbar, Anne des Rieux, Christine Wyns

**Affiliations:** 1 Gynecology-Andrology Unit, Institute of Experimental and Clinical Research, Medical School, Catholic University of Louvain, UCLouvain, 1200 Brussels, Belgium; federico.delvento@uclouvain.be (F.D.V.); jonathan.poels@uclouvain.be (J.P.); vermeulen.maxime@live.be (M.V.); maria.giudice@uclouvain.be (M.G.G.); marc.kanbar@uclouvain.be (M.K.); 2 Advanced Drug Delivery and Biomaterials Unit, Louvain Drug Research Institute, Catholic University of Louvain, UCLouvain, 1200 Brussels, Belgium; bernard.ucakar@uclouvain.be (B.U.); anne.desrieux@uclouvain.be (A.d.R.); 3 Department of Gynecology-Andrology, Saint-Luc University Hospital, 1200 Brussels, Belgium

**Keywords:** testicular tissue transplantation, fertility preservation, VEGF, PDGF, vascular maturity, necrosis inhibitor, spermatogonia stem cells, nanoparticles, tissue engineering

## Abstract

Avascular transplantation of frozen–thawed testicular tissue fragments represents a potential future technique for fertility restoration in boys with cancer. A significant loss of spermatogonia was observed in xeno-transplants of human tissue most likely due to the hypoxic period before revascularization. To reduce the effect of hypoxia–reoxygenation injuries, several options have already been explored, like encapsulation in alginate hydrogel and supplementation with nanoparticles delivering a necrosis inhibitor (NECINH) or VEGF. While these approaches improved short-term (5 days) vascular surfaces in grafts, neovessels were not maintained up to 21 days; i.e., the time needed for achieving vessel stabilization. To better support tissue grafts, nanoparticles loaded with VEGF, PDGF and NECINH were developed. Testicular tissue fragments from 4–5-week-old mice were encapsulated in calcium-alginate hydrogels, either non-supplemented (control) or supplemented with drug-loaded nanoparticles (VEGF-nanoparticles; VEGF-nanoparticles + PDGF-nanoparticles; NECINH-nanoparticles; VEGF-nanoparticles + NECINH-nanoparticles; and VEGF-nanoparticles + PDGF-nanoparticles + NECINH-nanoparticles) before auto-transplantation. Grafts were recovered after 5 or 21 days for analyses of tissue integrity (hematoxylin–eosin staining), spermatogonial survival (immuno-histo-chemistry for promyelocytic leukemia zinc finger) and vascularization (immuno-histo-chemistry for α-smooth muscle actin and CD-31). Our results showed that a combination of VEGF and PDGF nanoparticles increased vascular maturity and induced a faster maturation of vascular structures in grafts.

## 1. Introduction

Fertility impairment induced by anticancer treatments in the pediatric population [1,2,3,4,5] represents a major concern for the quality of life of oncological survivors [6,7], and several fertility preservation (FP) programs have been set up worldwide to preserve patients’ reproductive potential [8,9,10]. While for female and post-pubertal male patients FP is at a clinical stage [11,12,13], for pre-pubertal boys it is still considered experimental.

As spermatogonia are the only germ cells present in the testes before puberty [14], their cryopreservation is the only option for FP [15,16], with the perspective of either cell/tissue auto-transplantation [17] or in vitro maturation using different culture systems [18,19,20].

Although there is so far no reported outcome for clinical human prepubertal testicular cells or tissue auto-transplantation, several preclinical investigations in animal species [17], including non-human primates [21,22], pave the way for future clinical implementation. As a proof of concept, auto-transplantation of frozen–thawed prepubertal rhesus macaques testicular tissue fragments of large size (9–20 mm^3^) allowed complete spermatogenesis and the generation of offspring [23]. With regard to human tissue, frozen–thawed immature testicular tissue (ITT) xeno-transplanted to nude mice allowed preservation of spermatogonia able to proliferate and initiate differentiation [15,16,24]. However, as testicular sampling aimed at FP is usually kept at a minimum to avoid potential harm caused by the procedure, only small amounts of testicular tissue are available for cryostorage and future use [4].

A persistent observation when small ITT fragments (1–10 mm^3^) were xenografted was the loss of a high proportion of spermatogonia [24,25], as high as 67% after 5 days of transplantation [26]. This could be explained by the phylogenetic distance between the mouse and human, but also by the avascular transplantation procedure as there is no surgical anastomosis to the host´s vascular system [15]. Tissue revascularization has been shown to occur spontaneously in the grafts where small capillaries connect to the recipient’s larger blood vessels assuring tissue survival [27]. However, as observed in ovarian tissue transplantation [28], before the development of a neo-vascularization, the graft is exposed to a period of hypoxia, which might be responsible for the reduction in germ cell numbers.

Hence, improving or accelerating the development of a mature vascular network in grafts could increase both tissue and spermatogonia survival. In this regard, enhancing tissue vascularization with vascular growth factors has been the goal of several studies involving testicular tissue transplantation [29,30].

Vascular endothelial growth factor (VEGF) is a polypeptide that initiates angiogenesis by inducing chemotaxis and proliferation of endothelial cells [31,32]. It has been used to support bovine ITT xeno-transplantation, increasing both the weight of the recovered grafts and the number of seminiferous tubules containing elongated spermatids [33]. More recently, human ITT fragments cultured with VEGF 5 days before being xeno-transplanted to nude mice also showed a better seminiferous tubule integrity in grafts [30].

Unlike ex vivo supplementation of vascular growth factors, systemic administration of molecules that increase angiogenesis calls for caution because of severe possible collateral effects, such as hypotension [34] or promotion of tumor angiogenesis [35]. To overcome these limitations, localized delivery using tissue engineering techniques has been considered [36,37].

In our previous study, embedding of mice testicular tissue in an alginate hydrogel loaded with dextran-chitosan nanoparticles (NPs) delivering VEGF supported short-term angiogenesis, leading to a significantly increased vascular surface 5 days post-transplantation. However, such beneficial effect disappeared after 21 days, suggesting that the newly generated vascular structures might lack vessel stability [29]. These results motivated follow-up investigations on novel approaches aiming at decreasing the hypoxic damage to testicular cells and tissue. In this regard, controlled local drug delivery with NPs loaded with a necrosis inhibitor improved spermatogonial survival in mice testicular tissue auto-grafts [38]. Accelerating revascularization and improving vascular maturity in grafts is another strategy. Amongst the several growth factors involved in the generation and maturation of blood vessels [39], platelet-derived growth factor (PDGF) is a candidate molecule to optimize angiogenesis, as it was shown to play a role in the stabilization of vascular structures [40]. However, it is still unclear which approach is superior, either acting on graft revascularization or protecting cells and tissue from hypoxic damage, or a combination of approaches, and which molecules are the most effective.

Therefore, the objective of this study was to explore and compare the effects of sustained and localized delivery of two vascular growth factors and a necrosis inhibitor on mouse testicular tissue grafts.

Poly(D,L-lactide-co-glycolide)/poly(ethylene glycol (PLGA/PLGA-PEG) was used to form nanoparticles containing either one vascular growth factor (VEGF or PDGF) or a necrosis inhibitor (NECINH). Each molecule was encapsulated individually in separate nanoparticles that were then combined in the alginate hydrogel.

Testicular tissue from 4–5-week-old mice was obtained after bilateral orchidectomy, and fragments of 1 mm^3^ were used for orthotopic transplantation experiments. Before grafting, tissue fragments were encapsulated in a matrix composed of 1% calcium-alginate hydrogel (control) or in a 1% calcium-alginate hydrogel supplemented with one or a combination of drug-loaded nanoparticles, including five different conditions, namely, VEGF-NPs (V group); VEGF-NPs and PDGF-NPs (V+P group); NECINH-NPs (N group); VEGF-NPs and NECINH-NPs (V+N group); and VEGF-NPs, PDGF-NPs and NECINH-NPs (V+P+N group). Grafts were recovered after 5 or 21 days for analyses on tissue integrity, spermatogonial survival and vascularization.

## 2. Results

### 2.1. Nanoparticle Characterization

The physicochemical characteristics of the nanoparticles are summarized in Table 1.

The objective of this part of the study was to define how the different molecules were released from the NPs incorporated in a 1% alginate hydrogel and whether the combination of bioactive cues in the same hydrogel will impact their release.

**Figure 1:**
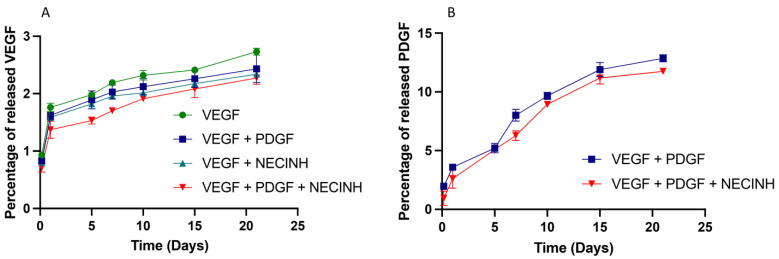
In vitro release of vascular growth factors from NPs incorporated alone or in combination with other NPs, in an alginate hydrogel. (**A**) VEGF-loaded PLGA:PLGA-PEG NPS and (**B**) PDGF-loaded PLGA:PLGA-PEG NPs were embedded in a 1% alginate matrix and VEGF and PDGF were quantified in the release buffer (40 mM CaCl_2_ PBS) at 34 °C for 21 days (*n* = 4).

VEGF release was very slow (less than 2.5% of the encapsulated dose in 21 days) and was not significantly impacted by the presence of other factors (Figure 1A). PDGF release in the same conditions was faster than VEGF (12% after 21 days) and was not affected by the presence of PDGF- or/and NECINH-loaded NPs (Figure 1B).

### 2.2. Tissue Integrity

The goal here was to evaluate the impact of NP supplementation on the integrity of an immature testicular tissue fragment orthotopically auto-transplanted after embedding in an alginate hydrogel.

Seminiferous tubule (ST) integrity was evaluated on hematoxylin–eosin-stained slides of grafts recovered after 5 (3231 STs) and 21 days (2181 STs).

**Figure 2:**
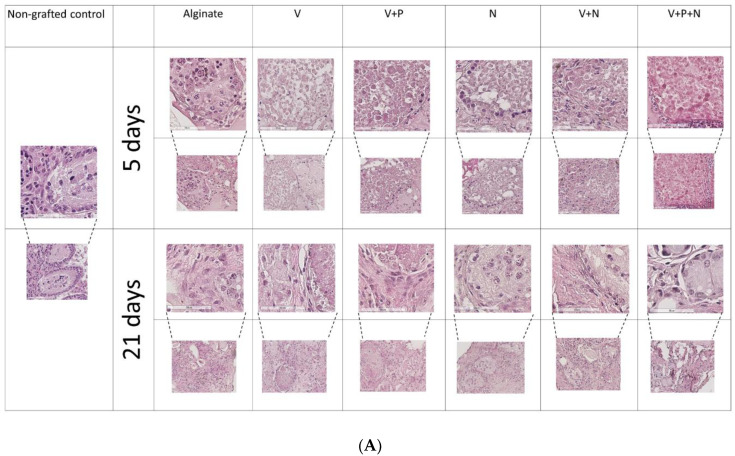

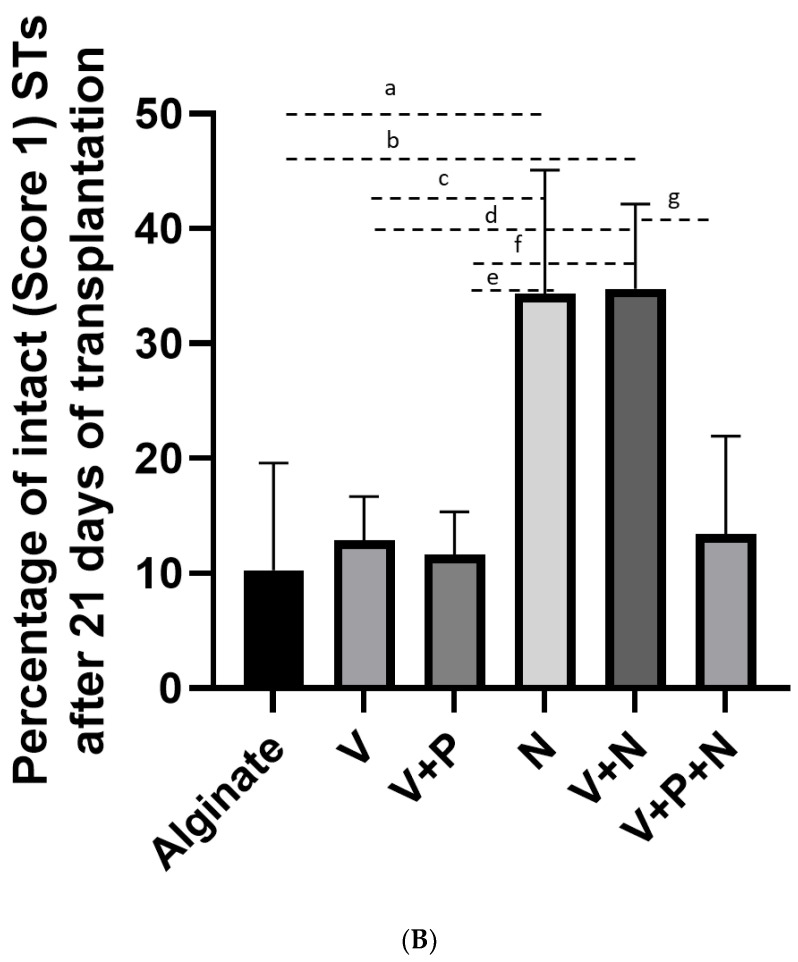
Impact of NP supplementation on seminiferous tubule integrity. Mouse testicular tissues were encapsulated in alginate supplemented with five different combinations in addition to a negative control (Alginate, V, V+P, N, V+N and V+P+N) for 21 days. Seminiferous tubule integrity was (**A**) visualized by hematoxylin–eosin-staining 5 and 21 days after orthotopic auto-transplantation and (**B**) quantified after 21 days. Results are expressed as the mean percentage of intact (Score 1) STs ± standard deviation. a: *p* = 0.02; b: *p* = 0.02; c: *p* = 0.04; d: *p* = 0.04; e: *p* = 0.03, f: *p* = 0.03; g: *p* = 0.04 (*n* = 3).

After 5 days of transplantation, the majority of the STs were classified as satisfactory (Score 2) based on morphological evaluation. No statistically significant difference between supplementations was observed when intact (Score 1) STs or intact and satisfactory (Score 1 + 2) STs were taken in account (Figure 2A and Table 2).

With regard to grafts recovered after 21 days, supplementation with NECINH-NPs (N) and the combination of VEGF and NECINH-NPs (V+N) were the most efficient at supporting the preservation of ST integrity compared to the other conditions (Figure 2A,B). Addition of PDGF-NPs had no additional effect when combined with VEGF-NPs alone (V+P) or with the combination of VEGF and NECINH-NPs (V+N+P).

### 2.3. Spermatogonial Survival

**Figure 3:**
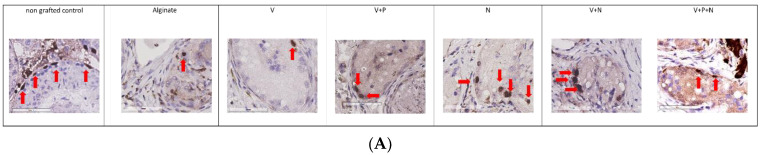

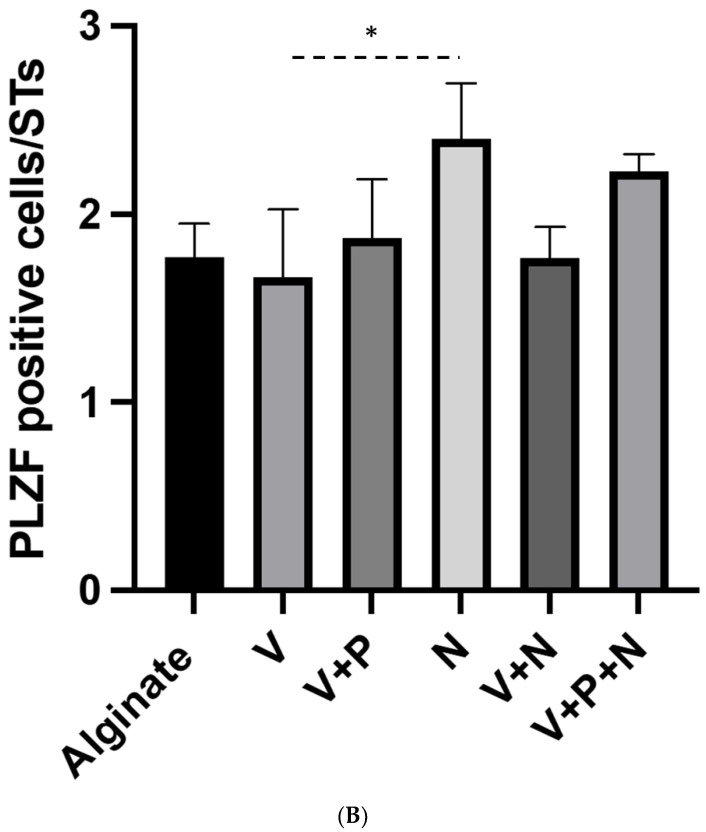
Impact of NP supplementation on germ cell survival 21 days after grafting. Mouse testicular tissue fragments were encapsulated in alginate supplemented with five different combinations in addition to a negative control (Alginate, V, V+P, N, V+N and V+P+N). (**A**) Immunostaining against PLZF was used to identify undifferentiated spermatogonia, (Red arrows highlight positive cells, scale bar = 60 μm) and (**B**) to quantify them. Results are expressed as the mean PLZF positive cells/STs (Alginate 1.77 ± 0.17; 1.79 ± 0.46; V+P: 1.87 ± 0.31; N: 2.4 ± 0.29; V+N. 1.76 ± 0.16; and V+P+N: 2.23 ± 0.08) (*n* = 3). * *p* = 0.03.

We focused on a specific population of undifferentiated spermatogonia that includes the spermatogonial stem cells using immunohistochemistry against promyelocytic leukemia zinc finger (PLZF) for their quantification in grafts (Figure 3A,B).

The number of PLZF-positive cells per ST after 21 days of transplantation was the highest when NECINH-NPs were added to the graft and was significantly higher than in grafts supplemented with VEGF-NPs. No significant difference was observed between the other conditions.

### 2.4. Vascularization

As neovascularization is crucial for graft survival and functionality, the impact of NP drug delivery on α-smooth muscle actin (α-SMA)-positive vessels was evaluated, as well as on stable blood vessels (identified by CD-31, a cell marker of mature endothelial structures). The vascular surface was quantified by morphometry using ImageScope on digitalized images of tissue recovered after 5 and 21 days of transplantation. Results are expressed as the measured surface of α-SMA-positive or CD-31-positive blood vessels per total graft surface ± standard deviation.

**Figure 4:**
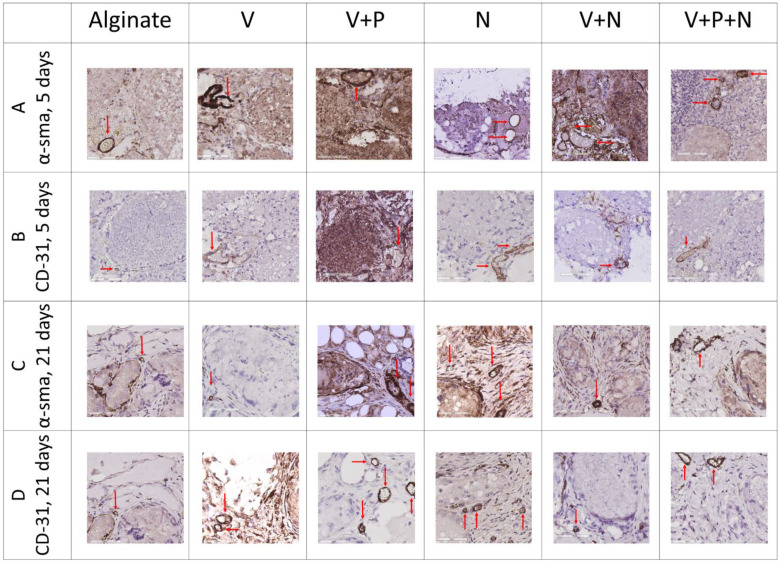
Impact of NP supplementation on the vascularization of mice testicular tissue auto-transplanted for 5 and 21 days. Mouse testicular tissue fragments were encapsulated in alginate supplemented with five different combinations in addition to a negative control (Alginate, V, V+P, N, V+N and V+P+N) and the grafts were stained after 5 and 21 days for α-SMA and CD31. Red arrows highlight positive vascular structures. Scale bar = 60 μm. *n* = 3.

**Figure 5:**
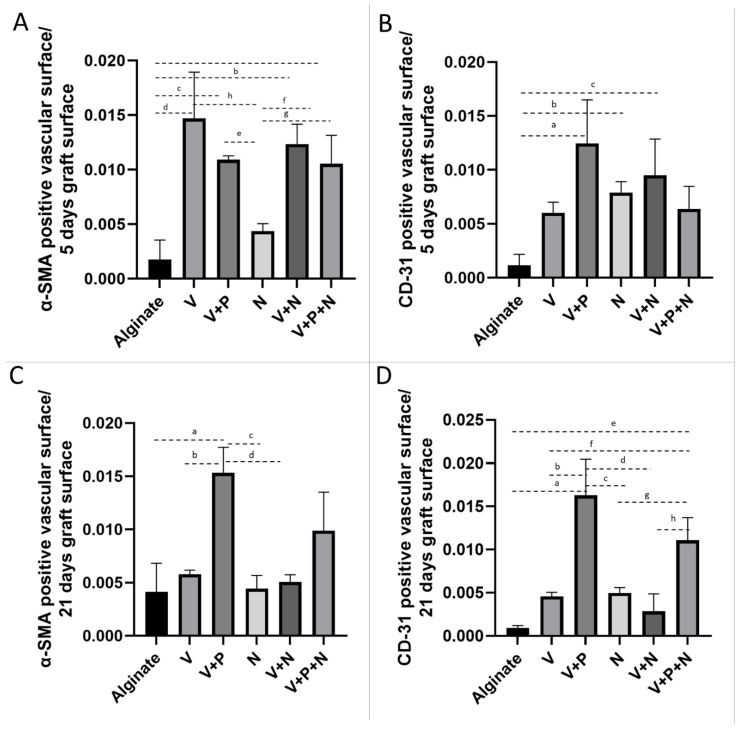
Impact of NP supplementation on the vascularization of mice testicular tissue auto-transplanted for 5 and 21 days. Mouse testicular tissue fragments were encapsulated in alginate supplemented with five different combinations in addition to a negative control (Alginate, V, V+P, N, V+N and V+P+N) and the positive surface for α-SMA (A and C, 5 and 21 days, respectively) and CD31 (B and D, 5 and 21 days, respectively) was quantified using VisioPharm (*n* = 36). (**A**) IHC for α-SMA, 5 days. Encapsulation with a matrix containing VEGF-NPs (alone or combined with PDGF-NPs or NECINH-NPs) increased vascular surfaces. Results are expressed as the mean positive vascular surface per total graft surface ± standard deviation: Alginate (1.74 × 10^−3^ ± 1.81 × 10^−3^); V (1.47 × 10^−2^ ± 4.21 × 10^−3^); V+P (1.09 × 10^−2^ ± 3.29 × 10^−4^); N (4.63 × 10^−3^± 6.85 × 10^−4^); V+N (1.23 × 10^−2^ ± 1.80 × 10^−3^); V+P+N (1.05 × 10^−2^ ± 2.64 × 10^−3^). a: *p* < 0.01; b: *p* < 0.01; c: *p* < 0.01; d: *p* < 0.01; e: *p* < 0.01; f: *p* = 0.01; g: *p* = 0.05; h: *p* < 0.01. (**B**) IHC for α-SMA, 21 days. Encapsulation with a matrix containing VEGF-NPs and PDGF-NPs increased vascular surface: alginate (4.13 × 10^−3^ ± 2.69 × 10^−3^); V (5.79 × 10^−3^ ± 3.92 × 10^−4^); V+P (1.53 × 10^−2^ ± 2.38 × 10^−3^); N (4.42 × 10^−3^ ± 1.25 × 10^−3^); V+N (5.06 × 10^−3^ ± 6.78 × 10^−4^); V+P+N (9.89 × 10^−3^ ± 3.62 × 10^−3^). a: *p* < 0.01; b: *p* < 0.01; c: *p* < 0.01; d: *p* < 0.01. (**C**) IHC for CD-31, 5 days. Supplementation with V+P, N and V+N improved vascular maturity compared to alginate encapsulation only. Alginate (1.04 × 10^−4^ ± 1.17 × 10^−3^); V (7.15 × 10^−3^ ± 6.00 × 10^−3^); V+P (1.20 × 10^−2^ ± 1.24 × 10^−2^); N (6.83 × 10^−3^ ± 7.9 × 10^3^); V+N (6.06 × 10^−3^ ± 9.48 × 10^−3^); V+P+N (8.63 × 10^−3^ ±6.37 × 10^−3^). a: *p* < 0.01; b: *p* = 0.0467; c: *p =* 0.01. (**D**) IHC for CD-31, 21 days. Supplementation with VEGF-NPs and PDGF-NPs increased vascular maturity regardless of NECINH-NPs (V+P and V+P+N). Alginate (9.27 × 10^−4^ ± 2.61 × 10^−4^); V (4.56 × 10^−3^ ± 4.82 × 10^−4^); V+P (1.63 × 10^−2^ ± 4.19 × 10^−3^); N (4.97 × 10^−3^ ± 6.27 × 10^−4^); V+N (2.84 × 10^−3^ ± 2.02 × 10^−3^); V+P+N (1.11 × 10^−2^ ± 2.64 × 10^−3^). a: *p* < 0.0001; b: *p* < 0.01; c: *p* < 0.01; d: *p* < 0.01; e: *p* < 0.01; f: *p =* 0.03; g: *p =* 0.04; h: *p* < 0.01. *n* = 3.

The α-SMA-positive vascular surface per graft increased when the graft was supplemented with VEGF-NPs, either alone or combined with other NPs, 5 days after transplantation (Figure 4A and Figure 5A). Combination with PDGF and NECINH had no significant effect. However, after 21 days, the surface of the α-SMA-positive blood vessels declined, except when the VEGF and PDGF NPs were combined (Figure 4C and Figure 5C).

A combination of VEGF- and PDGF-NPs, NECINH-NPs, and a combination of NECINH-NPs and VEGF-NPs led to a significant increase in CD31-positive surfaces in grafts (Figure 4B and Figure 5B) 5 days after transplantation. Only the addition of PDGF-NPs allowed to maintain a significantly larger surface of CD31 staining over a longer period (21 days) (Figure 4D and Figure 5D).

**Figure 6:**
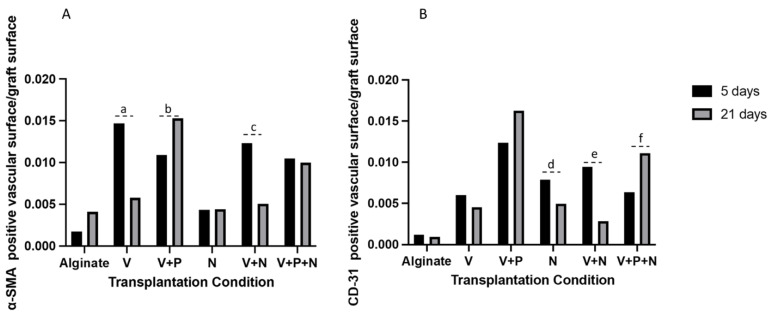
Impact of NP supplementation on the blood vessels’ evolution between 5 and 21 days in mice testicular tissue auto-transplants. (**A**) α-SMA- and (**B**) CD31-positive surfaces in the grafts were compared after 5 and 21 days of transplantation. a: *p =* 0.03; b: *p =* 0.04; c: *p* < 0.01; d: *p =* 0.01; e: *p =* 0.02; f: *p =* 0.03. *n* = 3.

When looking at the evolution over time of these vascular markers, an increased α-SMA-positive surface was recorded between 5 and 21 days when VEGF-NPs and PDGF-NPs were employed together (V+P group) (Figure 6A). The CD-31-positive vascular surface was increased in the V+P+N group between 5 and 21 days and reduced when the PDGF-NPs were not employed (N and V+N) (Figure 6B).

**Figure 7:**
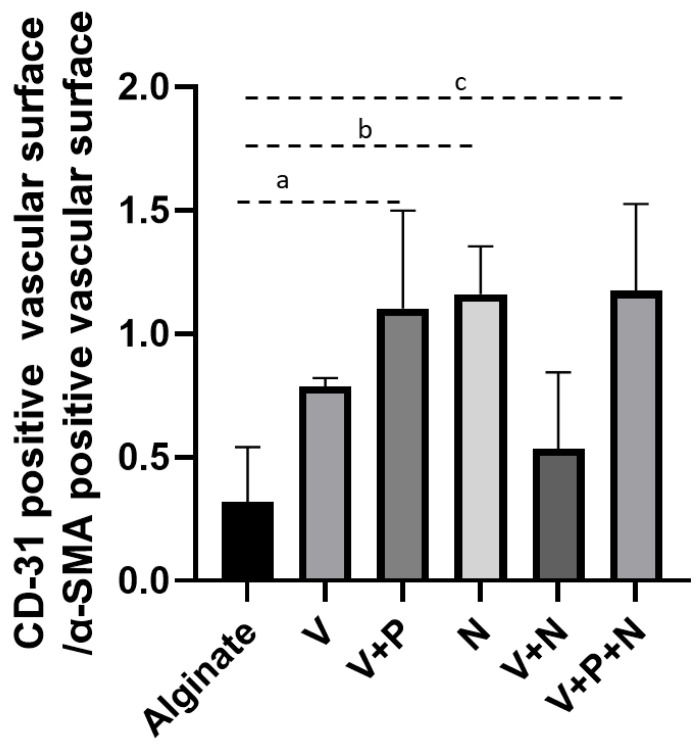
Impact of NP supplementation on neo-vascularization stabilization. The CD-31/α-SMA ratio was calculated for grafts retrieved after 21 days a: *p =* 0.04; b: *p =* 0.02; c: *p =* 0.02. *n* = 18.

The ratio between the CD-31/α-SMA-positive vascular surface, representing the proportion of stabilized vessels, was not different between groups after 5 days (Alginate: 2.44 ± 0.38; V: 0.42 ± 0.12; V+P: 1.14 ± 0.41; N: 1.84 ± 0.43; V+N: 0.79 ± 0.33; V+P+N: 0.61 ± 0.14. After 21 days, the proportion of stabilized vessels was statistically higher when the V+P, N and V+P+N groups were compared to the alginate group (Figure 7).

## 3. Discussion

Auto-transplantation of frozen–thawed testicular tissue is a potential option to restore a patient’s fertility after gonadotoxic treatment and could therefore play a role in improving the quality of life of prepubertal cancer survivors. Based on short- and long-term human ITT xeno-transplantation experiments, the reduction in spermatogonia numbers appeared to be the highest during the first three weeks following transplantation [15,24,26], a period of time that has been proven to be necessary for the generation of stable vascular structures [41] and that has been characterized by hypoxic damage to tissue and cells [42]. The optimization of the grafting technique, targeting an increased spermatogonial survival, represents an open challenge. Delivery of drugs directly to the transplanted tissue could reduce or avoid the potential collateral effects of a systemic administration and enhance the graft’s efficiency by improving tissue integrity and increasing the number of surviving spermatogonial stem cells able to differentiate into mature spermatozoa. Tissue engineering techniques that employ vascular growth factors and necrosis inhibitors could provide an essential support to small testicular fragments grafts.

Here, we showed that localized and sustained delivery of vascular growth factors from polymeric nanoparticles improved vascularization of mouse testicular tissue grafts.

Analysis of IHC for α-SMA on tissue recovered after 5 days of transplantation showed that VEGF-NPs increased the vascular surface in the graft. This result was not influenced by simultaneous supplementation with other types of NPs and it was not maintained after 21 days of grafting. While this is in agreement with previously published results, linking an increased short-term vascularization to NPs delivering VEGF [29], it motivated the addition in this study of a second growth factor known for its ability to stabilize neo-vascularization (PDGF) [40]. This led to further progress in terms of graft angiogenesis enhancement. Indeed, after 21 days of transplantation, both the vascular surface and vessel maturity were significantly increased by simultaneous addition of VEGF-NPs and PDGF-NPs, compared to alginate hydrogel and to supplementation with VEGF-NPs.

The reduction in vascular surface evidenced by α-SMA immunostaining observed after 3 weeks of grafting, when the only vascular growth factor administered was VEGF (V and V+N groups), can be linked to insufficient vessel stabilization. In support of this theory, addition of NPs delivering PDGF to VEGF-NPs (V+P group) was associated with an increased graft vascular surface at 21 days. Improvement in the CD-31/α-SMA ratio observed after 21 days with PDGF-NPs application (V+P and V+P+N groups) further corroborated our hypothesis that the increased vascular surface was due to an improved maturation of the vessels. Interestingly, vascular maturity also improved after 5 days of transplantation (CD-31 IHC) in the presence of PDGF, which suggests that adding PDGF-loaded NPs accelerated the revascularization process in grafts.

Two of the transplantation conditions involving NECINH-NPs (V+N and N group) were also associated with an improvement in short-term vascular stability. However, this effect was not maintained after 21 days unless PDGF-NPs were present.

Moreover, we observed a significant improvement in the CD-31-positive vascular surface between 5 and 21 days when NECINH was added to vascular growth factors (V+P+N group versus V+P group), which suggests that NECINH might support the effect of PDGF. A higher CD-31/α-SMA ratio in the V+P+N group after 21 days of grafting provided further support to this hypothesis. No previous report has linked NECINH to pericytes chemotaxis. However, necrosis inhibition and reduction of the inflammation process associated with NECINH administration [43] could exert a yet partially unknown effect on angiogenesis that could explain the observed improvement in vascular maturity. This lack of information on the potential role of NECINH in neovascularization therefore calls for further studies.

Simultaneous use of NPs containing vascular growth factors and NECINH (V+P+N group) did not result in an improvement in the intact (Score 1) seminiferous tubule sections number, nor in an improved spermatogonial survival compared to the other groups employing NECINH-NPs. Unknown interactions between these molecules or the negative effects of vascular growth factors on the STs’ integrity are therefore not excluded.

No previous study investigated the concurrent use of these bioactive molecules. PDGF plays a role in the stabilization of vascular structures, both in physiological situations and pathological processes, such as wound healing and cancer [44]. The effect on vessel maturity has been linked to the PDGF chemotactic properties on pericytes [40,45]. Potentially, PDGF could be responsible for increased ST fibrosis [46], which counteracts the beneficial effects of NECINH. This could explain our observations on tissue integrity after concomitant supplementation of the three types of NPs (V+P+N group), while this parameter was still improved in the N and V+N conditions.

Moreover, cell signal pathways involving PDGF have also been identified in several tissues of the human body, including the testis, with effects displayed especially during testicular embryonic development [47]. Hence, while the repercussion of PDGF delivery to support testicular tissue grafts has never been studied, its impact could likely go beyond angiogenesis.

It is important to mention that the in vitro release of vascular growth factors is not to be held accountable for any of the differences between groups, as the drug-delivery profiles were similar for all matrixes applied and were not influenced by the concurrent supplementation of multiple nanoparticles. NECINH release from NPs and alginate hydrogels already has been reported in a previous publication [38]. As combining different nanoparticles within the same gel did not impact their release, we assumed it would be the same for NECINH.

In addition, modification in the release profile of VEGF did not seem to change the vascular effects of the VEGF-NPs. Indeed, while the VEGF concentration in the alginate hydrogel was the same as the one used in our previous transplantation experiment [29], the rate of VEGF release was slower in this study (2.5% after 21 days with PLGA/PLGA-PEG NPs compared to 7.8% with Dextran-chitosan-NPs, reported by Poels et al. in 2016); still, the VEGF effect on neo-vascularization was similar.

ST integrity and spermatogonial survival after 21 days of transplantation were increased when nanoparticles containing NECINH were administered alone, corroborating previous results [38], although no further benefit was obtained by improving the vascular network in grafts with vascular growth factors. VEGF-regulated pathways within the testes could be implicated. Indeed, VEGF receptors have been found on testicular cells, including spermatogonia, Sertoli cells and Leydig cells [48], and an impact of VEGF on spermatogonial self-renewal and differentiation has been suggested, of specific importance being the balance between the different isoforms of VEGF [49]. We used VEGF-164 in our experiments based on the long-known role of this isoform in the angiogenesis process [31]. While VEGF-164 was shown to participate in germ cells proliferation [50], it has also been linked to increased germ cell differentiation [51]. Hence, the number of PLZF-positive cells could have been influenced by a triggered differentiation of germ cells within grafts. Further studies on the short-term impact of vascular growth factors on germ cells should be conducted to fully elucidate the underlying mechanisms.

Altogether, our results represent a step towards a betterment of the testicular tissue transplantation technique with the potential for clinical application. So far, use of vascular growth factors in clinical trials led to encouraging results when administered via a local injection, e.g., in the infarcted heart [52], although application outside of experimental settings is not available yet. Considering unwanted systemic effect in the context of cancer patients, sustained and localized drug delivery seems to be a better option for administration of such molecules. Delivery of VEGF through nanoparticles has also been explored in vivo in rodents, with promising results for pathological conditions such as limb ischemia [53]. As PLGA is an FDA-approved excipient [54] with possible applications in humans [55], the strategy explored in these experiments could potentially be applied in clinical trials. Moreover, other types of avascular transplantation procedures, involving, for example, skin [56] or ovarian tissue [57], could benefit from accelerated vascular maturity.

Overall, the role of locally controlled vascular growth factor administration and the effects of simultaneous multiple bioactive molecule delivery needs to be further elucidated, especially with regard to longer-term grafting of testicular tissue and the effects on germ-cell differentiation.

## 4. Materials and Methods

### 4.1. Encapsulation of Bioactive Factors in Polymeric Nanoparticles

Recombinant murine VEGF-164 was produced as previously described [58], while PDGF-BB (101–14 B Peprotech, London, UK) and necrosis inhibitor NecroX-5 were purchased (ALX-430–167-M005, Enzo life science, New York, NY, USA).

VEGF, PDGF and Necrox were encapsulated as previously described [38,59]. Briefly, for vascular growth factors, 50 mg of polymers (25 mg of Poly(D,L-lactide-co-glycolide) (PLGA) (Resomer^®^ RG 502H, Sigma-Aldrich-719897, Darmstadt, Germany) and 25 mg of PLGA/poly(ethylene glycol (PLGA-PEG)) (Resomer^®^ RGP d 50155, Boehringer Ingelheim, Ingelheim, Germany) were dissolved in 1 mL of dichloromethane. In total, 50 µg of VEGF (1 mg/mL) or PDGF (1 mg/mL) were incorporated into the formulation and encapsulated by double emulsion.

NECINH nanoparticles were produced by the single emulsion technique, as previously reported [38]. In total, 250 µg of NECINH were used for this formulation.

#### 4.1.1. Nanoparticle Characterization

Nanoparticles were characterized in terms of size, PDI (polydispersity index) and zeta-potential using a Zetasizer (Malvern Panalytical, Malvern, UK) [38].

To calculate the encapsulation efficiency, non-encapsulated VEGF and PDGF concentrations were determined by the ELISA (enzyme-linked immunosorbent assay) sandwich test (900-K99 and 900-K04, Peprotech, respectively).

#### 4.1.2. In Vitro Drug Release Profile

In vitro release experiments were performed for each formulation and different combinations.

For each condition, NPs were mixed with a solution of 1% alginate (SLM100, FMC BioPolymers, NovaMatrix™, Sandvika, Norway) in 3-(*n*-morpholino) propanesulfonic acid, 4-morpholinepropanesulfonic acid (MOPS buffer) (M3183; Sigma-Aldrich, Darmstadt, Germany). Then, 5 µL of CaCl_2_ (50 mM solution in MOPS) were added to 45 uL of alginate to form the gel. Hydrogels were covered with 250 µL of 40 mM CaCl_2_ in PBS and incubated at 34 °C for 21 days. Release media were collected and replaced at 4 h, and 1, 5, 7, 10, 15 and 21 days. The VEGF and PDGF concentrations were determined by ELISA. Results were expressed as the percentage of released growth factor compared to the total amount of growth factor initially introduced into the hydrogel.

### 4.2. Testicular Tissue Collection

Ethics Review Board and the Committee on Animal Research of UCLouvain (project 2018/UCL/MD/20, approval date: 22 June 2018) approved all experiments involving animals, and the laws currently in force in Belgium (Royal Decree on the Protection of Experimental Animals 29 May 2013) were respected.

Thirty-six NMRI (Naval Medical Research Institute) male mice were purchased from Janvier Labs (Saint Berthevin Cedex, France) and kept in individual cages in the animal facility of UCLouvain (Linné building, UCLouvain, Woluwe Saint-Lambert, Belgium), where they received animal care according to university guidelines. Room temperature and humidity were checked every day and a usual night/day cycle was applied. Food and water were available ad libitum.

Surgery was performed in an equipped area of the animal house facility of the UCLouvain University. Intraperitoneal injection of medetomidine (1 mg/kg) (Domitor, Pfizer, Cambridge, MA, USA) and ketamine (75 mg/kg) (Anesketin, Eurovet, Heusden-Zolder, Belgium) was used to provide anesthesia, while buprenorphine (0.1 mg/kg) (Temgesic, Schering Plough, Kenilworth, NJ, USA) provided analgesia.

Through a scrotal incision, we performed a bilateral orchidectomy. For each animal, one fragment of 1 mm^3^ of the testicular tissue was used for transplantation and one fragment of the same size was kept as the non-grafted control.

### 4.3. Encapsulation of Tissue and NPs

Fragments and NPs were encapsulated in alginate hydrogels, as previously described [29,38]. Briefly, we added NP solutions to the 1% alginate hydrogels to explore the impact of six different types of supplementation (Alginate, V, V+P, V+N and V+P+N). The concentrations of the bioactive molecules in the hydrogels were 0.06 μg/μL for VEGF [29,60] and PDGF [61], and 0.13 μg/μL for NECINH [38]. Mice testicular tissue fragments were placed in 45 μL of the hydrogel and 5 μL of CaCl_2_ were added to obtain gelation.

### 4.4. Testicular Tissue Transplantation

Hydrogels containing tissue fragments were orthotopically autografted and we reversed anesthesia with intraperitoneal injection of atipamezole (1 mg/kg) (Antisedan, Pfizer, Cambridge, MA, USA). After 2 different timings (5 and 21 days), mice were euthanized by cervical dislocation to allow graft recovery.

### 4.5. Tissue Analyses

Recovered grafts were kept in modified Davidson’s fluid (mDF) for one night before fixation in paraffin. Blocks were cut in 5 μm-thick sections that were placed on Superfrost Plus slides (VWR, Leuven, Belgium). Immunohistochemistry analysis were performed according to common protocols. After dewaxing and rehydration, slides were incubated for 30 min at room temperature (RT) in 0.3% H_2_O_2_ to block endogenous peroxidase activity and soaked in citrate buffer at 98 °C 60 min for heat-induced antigen retrieval. Then, 10% normal goat serum (NGS, Invitrogen, Merelbeke, Belgium) and 1% bovine serum albumin (BSA) (Invitrogen, Merelbeke, Belgium) were used (30 min at RT) to block non-specific binding sites.

Incubation with primary antibody followed indications and concentrations of the manufacturer’s datasheet. Anti-promyelocytic leukemia zinc finger (PLZF) (rabbit anti-PLZF antibody, 1/400, Sigma-Aldrich, HPA001499, St. Louis, MO, USA) was employed to identify undifferentiated spermatogonia [62]. Vascular structures and mature vascular structures were labeled, applying antibodies directed, respectively, towards α-SMA (anti-alpha smooth muscle actin, ab5694, Abcam, Cambridge, UK) [63] and CD31 (Cluster of differentiation-31) (PECAM-1/D8V9E- XP Rabbit, Cell Signaling, Danvers, MA, USA) [64]. Slides were incubated for 60 min at RT with a secondary anti-rabbit antibody (Envision+ system-labeled polymer-horseradish peroxidase (HRP); DAKO, K4003, Agilent, Santa Clara, CA, USA). Diaminobenzidine (DAKO K3468) was employed as chromogen, and Mayer’s hematoxylin was applied to counterstain the nuclei. Slides were washed with tap water and dehydrated before being sealed with Entellan mounting medium (Sigma-Aldrich, St. Louis, MO, USA) and coverslips. A Leica SCN400 slide scanner (Leica Biosystems, WETZLAP, Nußloch, Germany) was used to obtain digital images of the whole sections for tissue analyses.

The tissue integrity score was assessed on hematoxylin–eosin (HE)-stained slides, applying the following scoring criteria: STs were classified as intact (Score 1, cells adhere to the basement membrane, no signs of necrosis were spotted and cells cohesion was present), satisfactory (Score-2, intra-tubular cells could still be spotted in spite of presence of intra-tubular necrosis) or damaged (Score 3, STs showed complete necrosis). For PLZF IHC, results were expressed as the mean number of positive cells per seminiferous tubule (ST) section.

The α-SMA and CD31 results were expressed as the identified vascular surface per total graft surface

### 4.6. Statistical Analysis

Statistical analysis was performed using JMP Software (version JPM-pro 15, Cary, NC, USA).

Normal distribution of the data was evaluated with a Shapiro–Wilk test, and a one-way ANOVA with a Tukey post-hoc test was applied to compare the effects of the six encapsulation conditions. Student’s *t*-test was applied to assess the difference for each group at the two time points. Results are expressed as the mean ± standard deviation and *p* values < 0.05 were considered significant.

## 5. Conclusions

Supplementation of alginate hydrogels with nanoparticles delivering PDGF induced improvement in terms of vascularization and vascular maturity in testicular tissue grafts compared to supplementation with VEGF alone, while potential interactions with NECINH deserve further investigations.

Our study shows how support to cells and tissue can be provided by simultaneous local delivery of multiple bioactive molecules that target different biological processes. The results obtained with NP drug-delivery systems offer new perspectives for further application in regenerative medicine, especially in the field of fertility preservation.

## Figures and Tables

**Table 1:** Physicochemical characteristics of the NPs.

	Size (nm)	PDI	Z-Potential (mV)	Encapsulation Efficiency
VEGF-NPs	199 ± 18	0.18	−37 ± 7	90% ± 4%
PDGF-NPs	191 ± 8	0.14	−42 ± 9	94% ± 2%
NECINH-NPs	310 ± 13	0.26	−27 ± 5	65% ± 5%

**Table 2:** Impact of NP supplementation on seminiferous tubule integrity 5 days after grafting.

	Condition	1% Alginate (Control)	V	V+P	N	V+N	V+P+N
	
Intact (Score 1)		0.65% ± 0.71%	0.10% ± 0.17%	0.58% ± 0.61%	0.14% ± 0.25%	0.13% ± 0.23%	0%
Satisfactory (Score 2)		59.51% ± 15.91	50.70% ± 10.4%	67.21% ± 6.00%	62.60% ± 11.10	68.31% ± 3.30%	62.11% ± 3.81%
Damaged (Score 3)		38.70% ± 16.52%	48.79% ± 10.43%	32.11% ± 5.71%	37.19% ± 10.91	31.53% ± 3.60%	37.90% ± 3.81%

Results are expressed as the mean percentage of STs ± standard deviation.
